# Unveiling STRs instability in a colorectal cancer FFPE sample: a case report

**DOI:** 10.1007/s00414-024-03341-w

**Published:** 2024-10-08

**Authors:** Giulia Soldati, Chiara Saccardo, Dario Raniero, Domenico De Leo, Stefania Turrina

**Affiliations:** Department of Diagnostics and Public Health, Section of Forensic Medicine, Forensic Genetics Lab, University of Verona, Verona, Italy

**Keywords:** Instability of microsatellites, Loss of heterozygosity, Cancerous tissues, Formalin fixed paraffin embedded tissues, Forensic genetics profiles, Case report

## Abstract

In forensic genetics, sometimes formalin-fixed paraffin-embedded (FFPE) biopsy material taken during life is the only biological sample available for individual identification or paternity testing. In most cases, this biological tissue is characterized by the presence of tumor cells characterized by instability and loss of heterozygosity of microsatellites (MSI/LOH) compared to the DNA present in cells of normal tissue.In this case report, two FFPE samples from the same male subject were available for genetic investigation: one sample with colorectal cancer tissue and the other with normal tissue (no cancerous histopathological features). The comparison of the genetic profiles obtained from DNA extracted from the two tissues showed in the tumor tissue the presence of three genomic instability phenomena affecting FGA, CSF1P0, D21S2055 loci, located on three distinct autosomal chromosomes, and one duplication phenomenon affecting the DYS438. Therefore, due to the MSI/LOH phenomena, the genetic profile acquired from the tumor tissue was distorted and thus generated a fictitious genetic profile, not corresponding to the subject’s real one (normal tissue free of tumor cells).

## Introduction

When no other material is available, archival pathology specimens such as formalin-fixed paraffin-embedded (FFPE) tissues can be used for forensic individual identification or paternity testing. However, as in most cases, these tissues were stored following biopsies performed to check for the presence of cancer cells and identify cancer typology, alterations of short tandem repeats (STRs) used in the forensic field are possible [1]. It is well-documented that cancer tissues may show microsatellite instability (MSI) when there is the accumulation of somatic alterations in the microsatellite’s length and loss of heterozygosity (LOH) when one or multiple alleles are lost [2–7]. The observation of MSI and LOH in a genetic profile can introduce significant problems in the interpretation of results, leading to false exclusions in both paternity tests and personal identification cases, especially in cases where mutational phenomena occur at more than two loci, as in this specific case report. Different types of tumors and target organs are subject to these genomic alterations; an exceptionally high frequency of these phenomena is seen in colorectal carcinomas, gastrointestinal carcinomas, bladder tumors, and oral cancers. Colorectal cancer (CRC) is the third most common cancer in men and the third most common cause of cancer-related deaths in both men and women, representing a significant public health problem globally [8, 9]. According to current knowledge, CRC develops mainly through a gradual accumulation of genetic and epigenetic alterations in the genome, including widespread microsatellite instabilities.

In this case report, two FFPE samples collected from the same male subject were available for genetic investigation: one sample with CRC tissue and the other with no cancerous histopathological features. From a forensic perspective, the analysis of this casework confirms that great care must be taken when analyzing DNA typing results from tumor tissue samples.

## Materials and methods

Two FFPE samples collected from the same male subject underwent forensic genetic investigations: in the first sample, colorectal cancer cells were found by the pathologist, whereas in the second one, no cancer cells were detected.

Using a scalpel, excess paraffin was removed from the FFPE blocks; then, 3 slices of each FFPE block with a thickness of approximately 10 μm, obtained with a microtome, were used to extract the DNA. After the deparaffinization procedure with xylene and ethanol, carried out following the first part of the QIAGEN protocol [10], genomic DNA was isolated from the two FFPE samples using the QIAamp^®^ DNA Mini Kit (QIAGEN), followed by quantification with the Qubit Fluorimeter using the Qubit dsDNA HS Assay Kit (Thermo Fisher Scientific). Both DNA samples were normalized to a concentration of 1 ng/µl.

Five commercially available multiplex kits were used for the amplification of autosomal and gonosomal short tandem repeats (STRs): PowerPlex^®^ Fusion System, AmpFLSTR™ Identifiler™ PCR Amplification Kit, Investigator HDplex Kit, PowerPlex^®^ Y23 System, and Investigator Argus X-12 QS Kit.

Amplicons genotyping was performed with the SeqStudio™ Genetic Analyzer for HID (Applied Biosystems), using WEN Internal Lane Standard 500, GeneScan 500 LIZ, DNA Size Standard 550 (BTO) as internal standard, and specific allelic ladder provided by the manufacturers of each kit. Data were finally analyzed with the GeneMapper^®^ ID-X Software v1.6.

When different allelic patterns between normal and tumor tissue in one or more STR loci, the amplification and typing analysis were repeated twice.

To calculate the loss of heterozygosity the following equation was used [1], in which the allelic loss is given when the LOH value is less than 0.5 or higher than 2.0:

The entitled person’s written informed consent under Italian law n.219/2017 and the approval from the University of Verona’s research ethics committee review (CARU/CARP-12) was obtained for the analysis of these two samples.

## Results and discussion

The genomic DNA extracted from one healthy FFPE tissue and one FFPE tissue with suspected colorectal cancer cells was amplified using three autosomal STR and two gonosomal STR amplification kits.

All amplification kits generated complete genetic profiles for both samples analyzed.

Analysis using the PowerPlex^®^ Fusion System kit revealed two discordances between normal and tumor tissue in the CSF1PO and FGA markers. In the first case, a loss of heterozygosity was observed with the complete loss of allele 12 in the suspected colorectal cancer tissue; in the second case, a homozygous 21,21 genotype in the normal tissue was replaced by a heterozygous 21,22 genotype in the suspected colorectal cancer tissue. The same STR variants were confirmed with the AmpFLSTR™ Identifiler™ PCR Amplification Kit; the only difference was that there was no total loss of allele 12 but a pronounced imbalance of the two alleles. However, this imbalance still indicates a loss of heterozygosity since the application of Eq. 1 returned a LOH value of 3.87.

1 $${\rm{LOH = }}{{{{{\rm{peak}}\,{\rm{height}}\,{\rm{of}}\,{\rm{normal}}\,{\rm{allele2}}} \over {{\rm{peak}}\,{\rm{height}}\,{\rm{of}}\,{\rm{normal}}\,{\rm{allele1}}}}} \over {{{{\rm{peak}}\,{\rm{height}}\,{\rm{of}}\,{\rm{tumor}}\,{\rm{allele2}}} \over {{\rm{peak}}\,{\rm{height}}\,{\rm{of}}\,{\rm{tumor}}\,{\rm{allele1}}}}}}$$.

In addition, the autosomal chromosomes were also amplified with the Investigator HDplex Kit, which, having a different panel of markers from the previous multiplexes, showed an additional LOH at the D21S2055 locus, in which the allele 20.1 was utterly absent in the suspected colorectal cancer tissue.

A Y-STR duplication was finally evidenced at the DYS438 marker amplified with the PowerPlex^®^ Y23 System, in which the hemizygous allele 12 in the normal tissue showed up as a duplication with alleles 11 and 12 in the suspected colorectal cancer tissue.

No discrepancy emerged between the X-STR genetic profile obtained from the normal and CRC samples.

**Fig. 1:**
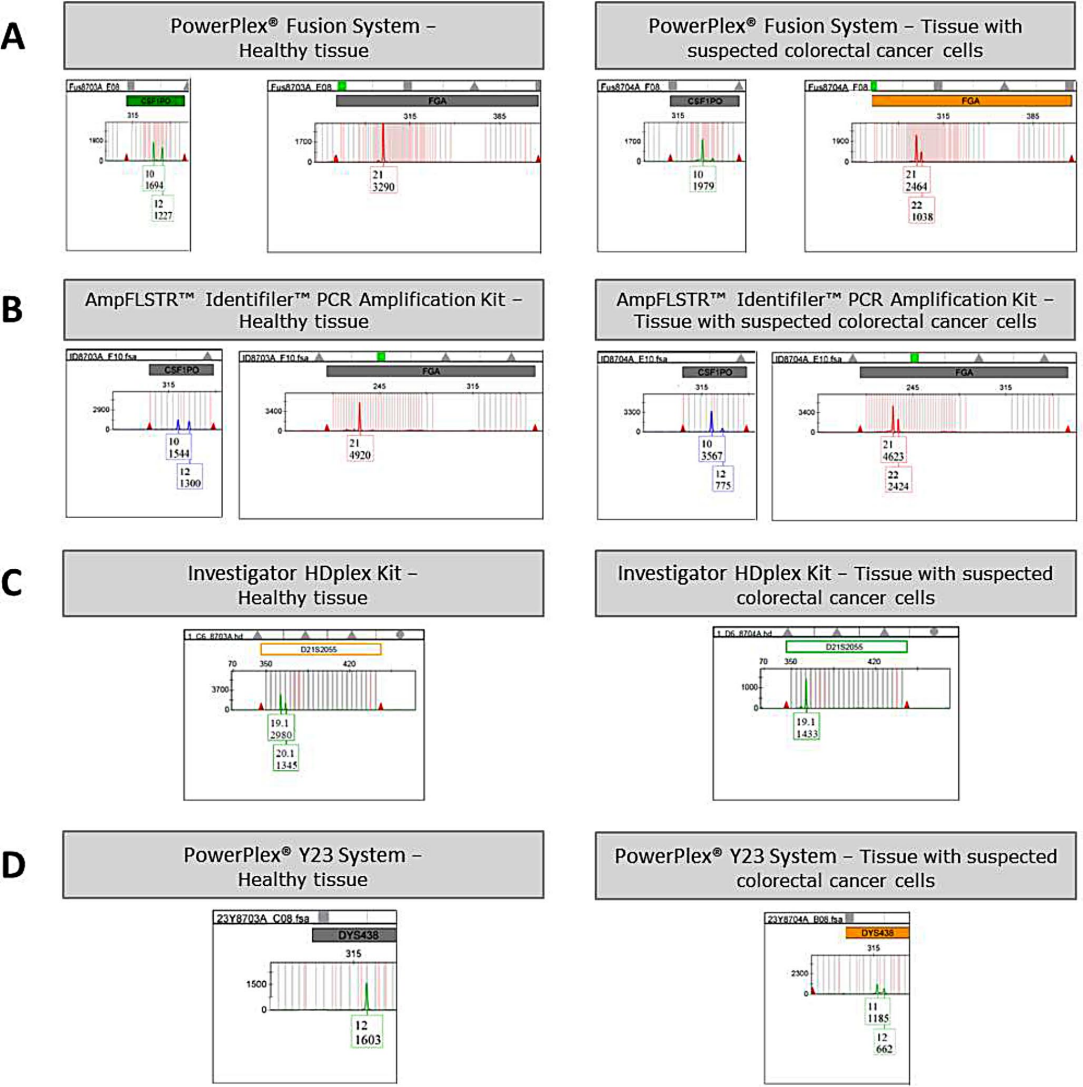
Electropherograms obtained with the different kits. **A**) Genotype alterations at CSF1PO and FGA markers obtained by amplification of healthy tissue and tissue with suspected colorectal cancer cells with the PowerPlex^®^ Fusion System; **B**) Genotype alterations at CSF1PO and FGA markers obtained by amplification of healthy tissue and tissue with suspected colorectal cancer cells with the AmpFLSTR™ Identifiler™ PCR Amplification Kit; **C**) Genotype alteration at D21S2055 locus obtained by amplification of healthy tissue and tissue with suspected colorectal cancer cells with the Investigator HDplex Kit; **D**) Genotype alteration at DYS438 locus obtained by amplification of healthy tissue and tissue with suspected colorectal cancer cells with the PowerPlex^®^ Y23 System

A summary of the results is shown in Table 1; Fig. 1.

**Table 1:**
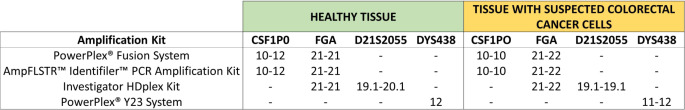
Alterations of short tandem repeats at CSF1PO, FGA, D21S2055, and DYS438 markers observed in the genetic profiles obtained from the healthy and suspected colorectal cancer tissues

Colorectal carcinoma is a malignant neoplasm arising from the epithelium of the intestine’s terminal tract, including the colon and rectum. It is a disease of high incidence and can be classified into various subtypes based on molecular characteristics. A relevant aspect in characterizing this tumor is microsatellite instability, which can be distinguished into two main categories: MSI-low and MSI-high [6]. The latter variant is generally associated with a less favorable prognosis, and current evidence suggests a reduced response to conventional chemotherapy. The phenomenon of microsatellite instability is extensively studied, particularly in the context of colorectal cancer, where MSI is present in about 8-20% of cases. The prevalence of MSI is higher in stage II (20%) than in stage III (12%) and stage IV (4%) colorectal cancer [11–13].

In clinical practice, the observation of MSI and LOH is an important prognostic and predictive factor for tumors; in contrast, in forensic genetics practice, in individual identification, the presence of MSI and LOH introduces significant problems in interpreting genetic profiles acquired from DNA extracted from tumor tissue, especially when the change in genotype, compared to the expected one, involves more than a single STR. To date, several studies have shown that using biopsy tissue characterized by tumor cells in forensic genetics investigations is questionable, mainly when dealing with gastrointestinal and colorectal tumors, which are more burdened by MSI and LOH phenomena [1, 3, 14]. Nevertheless, the literature does not contain many cases in which a single biological sample shows mutation and duplication phenomena affecting several autosomal and gonosomal chromosomes.

To the best of the Authors’ knowledge, this case report represents a unique contribution to the field of forensic genetics, as several panels of autosomal and gonosomal markers were analyzed in parallel, allowing the identification of four distinct chromosomes affected by genomic variations at STR loci. In comparison, Rubocky et al. [15] detected a loss of heterozygosity phenomenon limited to the D13S317 marker alone in a tumor sample; Zhou et al. [7] instead observed genotype alterations due to LOH in the D2S1338, D2S441 and D2S1776 markers, all located on chromosome 2. Furthermore, while MSI and LOH phenomena are repeatedly documented in the literature for the markers CSF1PO and FGA [1, 6, 14, 16], to the Authors’ knowledge, evidence at the marker D21S2055 is absent. This is presumably attributable to the fact that marker D21S2055 is amplified exclusively in the Investigator HDplex Kit marker panel, which includes highly polymorphic STR markers (D2S1360, D3S1744, D4S2366, D5S2500, D6S474, D7S1517, D8S1132, D10S2325, D12S391, D18S51, D21S2055, SE33). By including some additional markers not included in commonly used standard amplification kits, such a kit offers greater discriminatory capacity in complex cases.

Concerning the DYS438 locus, duplication phenomena related to alleles 11,12 were never described, except for two cases out of 50,859 reported by Jackie Johnson [17], detected on non-cancerous samples.

The duplication described in this study is not an intrinsic and distinctive duplication phenomenon of the subject under investigation but is closely linked to tumor cells in disease rather than healthy tissue. Therefore, this condition is relevant in evaluating a parental ascertainment between a father and a male child in which the same Y-STRs haplotype must be shared.

As mentioned above, loss of heterozygosity and microsatellite instability are relevant hallmarks in numerous tumors. These features are typically identified through the analysis of dinucleotide and mononucleotide markers. However, DNA instability can also affect other classes of short tandem repeated sequences, such as tetranucleotide repeats, routinely used in forensic and paternity testing [1].

In this study, due to the MSI/LOH phenomena, the genetic profile acquired from the tumor tissue was distorted from the reference one and thus generated a fictitious genetic profile, not corresponding to the subject’s real one (normal tissue free of tumor cells). If normal biopsy material had not also been available, there would have been no possibility of comparison to confirm the acquired genetic profile, and such a profile would have been used for individual identification or the search for biological paternity, for which the certainty of the exclusion of the parental relationship requires a minimum number of at least two STRs to exclude it, leading to the formulation of erroneous conclusions.

Unfortunately, the absence of reliable reference samples is a significant limitation; moreover, if only biopsy material containing tumor cells is available, individual identification and parental analysis may be inaccurate.

Therefore, in consideration of the high number of mutated markers on different chromosomes (three autosomal chromosomes and the Y chromosome), the scientific community must be aware of these potential abnormalities, as the increased use of human DNA for individual identification and parentage testing may necessitate clinical samples, such as biopsies, as reference material. Such samples require careful and rigorous handling, particularly when the presence of normal tissue within the material analyzed has not been confirmed.

## Conclusions

In forensic genetics, it is sometimes necessary to use formalin-fixed paraffin-embedded (FFPE) biopsy material to extract the DNA for individual identification or biological parental testing. Often, such material is stored in hospital histothecae following biopsy examinations of living patients to ascertain the presence or absence of cancer cells and, if positive, to identify their type. It is not uncommon for the biopsy material retained to test positive for tumor cells, while comparison material with healthy tissue is rarely present. Tumor genomic instability linked to forensic DNA markers, such as instability and loss of microsatellite heterozygosity, are frequently found in tumor tissues subjected to forensic genetics investigations. These phenomena significantly compromise the results of analyses, generating altered genetic profiles that hinder the correct interpretation of data.

It would therefore be desirable that before subjecting biopsy material of a suspected nature to forensic-genetic analysis, a histopathological investigation of the tissue should be carried out to isolate and eliminate the possible tumor component from the normal one, which remains the only component that can be used for forensic purposes.

## Data Availability

The data that support the findings of this study are not openly available due to reasons of sensitivity and are available from the corresponding author upon reasonable request.
